# Distribution status of genetically modified soybeans from the United States and Canada to Japan in 2021 and 2022

**DOI:** 10.1080/21645698.2024.2444048

**Published:** 2024-12-26

**Authors:** Keisuke Soga, Yu Hashimoto, Tomohiro Egi, Chie Taguchi, Satoko Yoshiba, Norihito Shibata, Kazunari Kondo, Reona Takabatake

**Affiliations:** a Division of Biochemistry, National Institute of Health Sciences, Kanagawa, Japan; b Division of Analytical Science, Food Research Institute, National Agriculture and Food Research Organization, Tsukuba, Ibaraki, Japan; c Food Labeling Monitoring Department, Food and Agricultural Materials Inspection Center, Saitama, Japan; d Department of Food Safety and Management, Faculty of Food and Health Sciences, Showa Women’s University, Setagaya-ku, Japan

**Keywords:** Distribution, event, genetically modified (GM), stacked variety, soybean

## Abstract

The number of authorized genetically modified (GM) soybeans has increased worldwide. In Japan, 34 GM soybeans containing single events and their stacked varieties have been approved as food. However, not all approved GM events are commercially cultivated or distributed. In this study, we evaluated domestically distributed samples from the United States (US) and Canada using 17 event-specific detection methods for GM soybeans. Identity-preserved (IP) soybean samples imported from the US and Canada, and non-IP samples from the US in 2021 and 2022 were analyzed. Four GM soybean events consisting of MON89788, A5547–127, MON87708, and DAS-44406 were detected in all lots in the non-IP samples. Furthermore, a single-kernel-based analysis was conducted to determine whether the detected GM soybean events are stacked. The results suggest that DAS-44406 is rapidly increasing, particularly as a single event among GM soybeans.

## Introduction

The global area under genetically modified (GM) crops is continually increasing and reached 206.3 million hectares in 2023.^1^ However, some consumers still express concerns about the utilization of genetically modified organisms (GMOs). In response, many countries and areas have passed laws requiring food labeling systems to indicate the presence of authorized GM crops. Under such conditions, not only the planted area, but also the number of varieties of GM events has been continuously increased. The main commercially grown GM crops are soybean, maize, cotton, and oilseed rape. In 39 countries and areas, 472 GM crops have been approved for food, feed, or environmental release.^2^

Soybeans are among the most important crops in Japan. The domestic consumption of soybeans as oil and food exceeds 4 million tons/y, but the self-sufficiency ratio for the crop is only 6%.^3^ Most of the soybeans consumed in Japan are mainly imported from the United States (US) and Canada where GM soybeans account for over 90% of the soybean cultivation area. In many countries including Japan, GM-labeling of food is mandatory if the GM content exceeds the authorized threshold level of the country. For example, in the European Union, Korea and the US, the threshold levels have been set at 0.9%, 3%, and 5%, respectively.^4,5^ In Japan, non-GM maize and soybeans are segregated and imported from other countries by an identity-preserved (IP) handling system.^6^ However, the unintentional commingling of GM products in non-GM materials is inevitable. In such cases, up to 5% unintentional commingling of approved GM maize or soybeans is generally accepted, and foods within this level of GM content can voluntarily be labeled as “handled to prevent commingling of GMO,” while non-GMO labeling is permitted when commingled GM contents are not detectable instead of 5%.^7–9^

The polymerase chain reaction (PCR) technique is widely used to detect and quantify GM crops in foods and feed. PCR detection methods can be largely classified into event-specific and screening methods. In event-specific detection, a unique sequence at the junction between the plant genome and recombinant DNA is used as the target. Screening methods target commonly conserved elements among many GM events, such as the Cauliflower Mosaic Virus 35S promoter (P35S), the nopaline synthase terminator (TNOS), and the 5-enolpyruvylshikimate-3-phosphate synthase (EPSPS).

In the Japanese standard method, qualitative detection methods for processed foods containing GM soybeans targeting MON89788, and P35S screening have been adopted.^10^ Additionally, quantification methods targeting GTS 40-3-2 (RRS), MON89788, and A2704–12 are used.^10^ In a previous study, non-IP soybean samples imported to Japan from the US and Brazil in 2017 were analyzed, revealing that the GM events, RRS, MON89788, and A2704–12 constituted the majority of GM events in these samples.^11^ In subsequent years, the number of approved GM events has been increasing in Japan. By the end of April 2024, 19 single GM soybean events and 15 stacked varieties had been approved.^12^ There are no surveys for distributed samples in recent years, despite the number of approved GM soybeans, which were not covered in the previous study, increased. In this study, we investigated IP and non-IP soybean samples imported from the US and Canada in 2021 and 2022 using 17 sets of event-specific real-time PCR detection methods. In case of large-scale distribution is revealed of GM event(s) other than the events or stacked varieties covered by the current standard method, it will be necessary to develop a detection method for those event(s).

## Materials and Methods

### Plant Materials

Non-IP and IP soybean samples from the 2021 and 2022 harvests were obtained from Japanese trading companies. These samples included five different non-IP and IP lots from the US and five different IP lots from Canada. The non-IP samples were imported for oilseeds processing, whereas the IP samples were imported for soy sauce production. Each sample was sourced from a different company, with non-IP samples labeled as N1, N2, N3, N4, and N5, and IP samples as I1, I2, I3, I4, and I5. The sample sizes ranged from 500 to 1500 g. Six certified reference materials (CRMs) in powder form were purchased from Sigma-Aldrich (St. Louis, MO, USA): RRS (Event 40-3-2), DP-305423 (ERM-BF426d), DAS-68416 (ERM-BF432d), DAS-44406 (ERM-BF436b), DAS-81419 (ERM-BF437b), and GMB151 (ERM-BF443b). Eleven CRMs were purchased from the American Oil Chemists’ Society (Urbana, IL): ground seeds of MON89788 (AOCS 0906-B), MON87701 (AOCS 0809-A), MON87705 (AOCS 0210-A), BPS-CV127 (AOCS 0911-C), MON87708 (AOCS 0311-A), MON87769 (AOCS 0809-B), MON87751 (AOCS 0215A), and SYHT0H2 (AOCS 0112A), and DNA extracts of A2704–12 (AOCS 0707-B), A5547–127 (AOCS 0707-C), and FG72 (AOCS 0610-A).

### DNA Extraction

Soybean genomic DNAs were extracted using a DNeasy Plant Maxi Kit (Qiagen, Hilden, Germany) and a GM quicker (NIPPON GENE Co., Ltd., Tokyo, Japan) according to the Japanese standard method.^10^ DNA extraction was performed in duplicate for each sample with the extracted DNA solutions labeled as follows: I1–1 and I1–2 for I1, and N1–1 and N1–2 for N1. The concentration and quality of the extracted DNA solutions were evaluated by measuring ultraviolet absorbance with a NanoDrop One spectrophotometer (NanoDrop Technologies, Wilmington, DE, USA). The concentration of the genomic DNA was adjusted to 10 ng/μL and used as a template in PCR analysis.

### Individual Kernel Detection Analysis

A kernel-based detection analysis was performed, as previously described.^11,13,14^ In each sample, 48 to 53 soybean kernels were prepared using a grain counter plate (For 100 Soybeans; Fuji Kinzoku, Tokyo, Japan). Each soybean kernel was individually ground using a Multi-beads Shocker (MB601NIHS; Yasui Kikai Co., Osaka, Japan), and DNA extraction from the ground kernels was performed using the GM quicker, following the manufacturer’s instructions.

### PCR Analysis

The primers and probes used in this study and the references are listed in Table 1.^15–27^ Oligonucleotide DNA primers and TaqMan probes were synthesized by FASMAC Co. Ltd. (Kanagawa, Japan) and Thermo Fisher Scientific (Carlsbad, CA), respectively. The probes were labeled with 6-carboxyfluorescein (FAM) and 6-carboxytetramethylrhodamin (TAMRA) at the 5′ and 3′ ends, respectively. The DAS-68416 and FG72 probes were labeled with minor groove binders at the 3′ end, and for the GMB151 and SYHT0H2 probes, black hole quenchers were used instead of TAMRA. The 25-μL reaction mixture contained 5 μL of the template DNA, 12.5 μL of the TaqMan Universal PCR Master Mix (Thermo Fisher Scientific), 0.5 μM primer pair, and 0.2 μM probe. The step-cycle program was set to 2 min at 50°C, 10 min at 95°C, 45 cycles of 30 s at 95°C, and 1 min at 59°C. The assay was repeated twice for each analysis using the ABI PRISM 7900HT (Thermo Fisher Scientific) or the LightCycler 480 (Roche Diagnostics).

**Table 1.** Primer and probe sequences.

Target		Sequences	References
RRS	RRS 01–5	CCTTTAGGATTTCAGCATCAGTGG	15
	RRS 01–3	GACTTGTCGCCGGGAATG	
	RRS-Taq	CGCAACCGCCCGCAAATCC	
MON89788	MON89788-F	TCCCGCTCTAGCGCTTCAAT	15
	MON89788-R	TCGAGCAGGAC CTGCAGAA	
	MON89788-P	CTGAAGGCGGGAAACGACAATCTG	
A2704–12	KVM175	GCAAAAAAGCGGTTAGCTCCT	15
	SMO001	ATTCAGGCTGCGCAACTGTT	
	TM031	CGGTCCTCCGATCGCCCTTCC	
A5547–127	KVM175	GCAAAAAAGCGGTTAGCTCCT	15
	SMO001	ATTCAGGCTGCGCAACTGTT	
	TM031	CGGTCCTCCGATCGCCCTTCC	
DP-305423	DP305-f1	CGTGTTCTCTTTTTGGCTAGC	15
	DP305-r5	GTGACCAATGAATACATAACACAAACTA	
	DP305-p	TGACACAAATGATTTTCATACAAAAGTCGAGA	
MON87701	MON87701 1	CGTTTCCCGCCTTCAGTTTAAA	16
	MON87701 2	TGGTGATATGAAGATACATGCTTAGCAT	
	MON87701	TCAGTGTTTGACACACACACTAAGCGTGCC	
MON87705	MON 87,705	TTCCCGGACATGAAGCCATTTAC	17
	MON 87,705	ACAACGGTGCCTTGGCCCAAAG	
	MON 87,705	AAGAGACTCAGGGTGTTGTTATCACTGCGG	
MON87769	MON 87,769	CATACTCATTGCTGATCCATGTAGATT	18
	MON 87,769	GCAAGTTGCTCGTGAAGTTTTG	
	MON 87,769 probe	CCCGGACATGAAGCCATTTACAATTGAC	
MON87708	MON87708	TCATACTCATTGCTGATCCATGTAG	19
	MON87708	AGAACAAATTAACGAAAAGACAGAACG	
	MON 87,708	TCCCGGACTTTAGCTCAAAATGCATGTA	
CV127	SE-127-f4	AACAGAAGTTTCCGTTGAGCTTTAAGAC	20
	SE-127-r2	CATTCGTAGCTCGGATCGTGTAC	
	SE-127-p3	TTTGGGGAAGCTGTCCCATGCCC	
DAS-68416	DAS-68416-4_3f5	GTACATTAAAAACGTCCGCAATGTGT	21
	DAS-68416-4_3r3	GTTTAAGAATTAGTTCTTACAGTTTATTGTTAG	
	DAS-68416-4_3p3	TTAAGTTGTCTAAGCGTCAATA	
DAS-44406	DAS-44406-5F	TTATTGTTCTTGTTGTTTCCTCTTTAGG	22
	DAS-44406-5 R	CCTCAATTGCGAGCTTTCTAATTT	
	DAS-44406-6-5p1	ATTCGGACCTCCATGATGACCTTACCGTT	
DAS-81419	DAS81419-f2	TCTAGCTATATTTAGCACTTGATATTCAT	23
	DAS81419-r1	GCTTCAAGATCCCAACTTGCG	
	DAS81419-p3	ATCAACAGGCACCGATGCGCACCG	
FG72	MAE071	AGATTTGATCGGGCTGCAGG	24
	SHA097	GCACGTATTGATGACCGCATTA	
	TM325	AATGTGGTTCATCCGTCTT	
GMB151	PRIM1040	TCAAATCAACATGGGTGACTAGAAA	25
	PRIM1041	CATTGTGCTGAATAGGTTTATAGCTATGAT	
	TM1789	CAGTACTGGGCCCTTGTGGCGCT	
SYHT0H2	FE08316-F	GGGAATTGGGTACCATGCC	26
	FE08317-R	TGTGTGCCATTGGTTTAGGGT	
	FE08318-P	CCAGCATGGCCGTATCCGCAA	
MON87751	MON 87,751 primer 2	CTAAATTGCTCTTTGGAGTTTATTTTGTAG	27
	MON 87,751 primer 1	GGCCTAACTTTTGGTGTGATGATG	
	MON 87,751 probe	TGACTGGAGATCTCCAAAGTGAGGGGAAA	
Le1	Le1n02–5′	GCCCTCTACTCCACCCCCA	15
	Le1n02–3′	GCCCATCTGCAAGCCTTTTT	
	Le1-Taq	AGCTTCGCCGCTTCCTTCAACTTCAC	

## Results

### Evaluation of Identity-Preserved-Handled Samples

We examined the commingling (s) of GM soybean in the IP handled samples from the US and Canada produced in 2021 and 2022 by targeting 17 event-specific detection methods. The events tested were listed in Table S1. The results of the US samples are summarized in Table 2. MON89788 was detected in all samples except I1 in 2021, and MON87708 was detected in all samples in 2022 and I4 in 2021, although the obtained Cq values were relatively high across the board. RRS and A5547–127 were detected in I1, I3 and I5 of the samples from 2022, and DAS-44406 was detected in sample I5 from 2022. DNA extraction was repeated twice for each sample, and no other GM events were reproducibly detected from either DNA extraction. In the Canadian samples, MON89788 was detected only in sample I1 from 2021 (Table S2).

**Table 2.** List of Cq* values obtained from identity-preserved samples from the United States.

		2021										2022									
GM event	PC	Sample no.										Sample no.									
		I1–1	I1–2	I2–1	I2–2	I3–1	I3–2	I4–1	I4–2	I5–1	I5–2	I1–1	I1–2	I2–1	I2–2	I3–1	I3–2	I4–1	I4–2	I5–1	I5–2
RRS	25.88	–	–	–	–	39.53	–	–	–	–	–	33.61	33.02	–	–	34.32	33.99	–	40.00	33.45	33.37
MON89788	25.02	–	–	32.80	33.04	34.06	33.59	31.91	32.03	41.14	40.61	31.10	30.58	37.72	34.46	31.44	30.87	32.56	32.19	36.81	36.57
A2704–12	23.19	–	–	–	–	–	–	–	–	–	–	–	–	–	–	–	–	–	–	–	–
A5547–127	23.80	–	–	–	–	–	–	–	–	–	–	33.77	33.77	–	–	34.52	33.94	–	–	37.04	36.86
DP-305423	30.82	–	–	–	–	–	–	–	–	–	–	–	–	–	–	–	–	–	–	–	–
MON87701	24.28	–	–	–	–	–	–	–	–	–	–	–	–	–	–	–	–	–	–	–	–
MON87705	24.51	–	–	–	–	–	–	–	–	–	–	–	–	–	–	–	–	–	–	–	–
MON87769	24.71	–	–	–	–	–	–	–	–	–	–	–	–	–	–	–	–	–	–	–	–
MON87708	24.51	–	–	–	–	–	–	32.41	32.77	–	43.80	34.10	33.88	36.52	33.87	32.07	31.84	33.31	32.40	35.83	35.92
CV127	24.45	–	–	–	–	–	–	–	–	–	–	–	–	–	–	–	–	–	–	–	–
DAS-68416	29.61	–	–	–	–	–	–	–	–	–	–	–	–	–	–	–	–	–	–	–	–
DAS-44406	26.93	39.50	–	–	–	–	–	–	–	39.46	–	–	–	–	–	–	–	–	–	32.42	32.04
DAS-81419	25.01	–	–	–	–	–	–	–	–	–	–	–	–	–	–	–	–	–	–	–	–
FG72	23.52	–	–	–	–	–	–	–	–	–	–	–	–	–	–	–	–	–	–	–	–
GMB151	25.89	–	–	–	–	–	–	–	–	–	–	–	–	–	–	–	–	–	–	–	–
MON87751	24.72	–	–	–	–	–	–	–	–	–	–	–	–	–	–	–	–	–	–	–	–
SYHT0H2	24.16	–	–	–	–	–	–	–	–	–	–	–	–	–	–	–	–	–	–	–	–
Le1	26.76	24.22	24.11	24.13	24.11	24.24	24.25	24.14	24.17	24.32	24.22	25.11	26.19	27.26	25.04	24.56	24.30	24.29	23.11	23.33	23.07

*Cq : quantification cycle for real-time PCR analysis.

To estimate the approximate comingling levels of GM soybeans, we quantified MON89788, which was detected in multiple samples with comparatively low Cq values relative to other events. A quantification method for MON89788 using a standard plasmid has been developed and validated.^28,29^ The limit of quantification (LOQ) was 0.5% for the quantification method. The contents of MON89788 were near or below the LOQ in all samples (data not shown).

### Evaluation of Non-Identity-Preserved Samples

Next, we tested the non-IP samples from the US in a similar manner (Table 3). GM soybeans were detected in all non-IP samples, and MON89788, A5547–127, MON87708, and DAS-44406 were detected in all samples. RRS was detected in samples N1, N3, N4, and N5 from 2021, and in samples N2, N4, and N5 from 2022. A2704–12 and MON87701 were only detected in samples N4 and N3 from 2022, respectively, whereas FG72 was detected in samples N1, N2, N3, and N5 from 2021, and samples N3, N4, and N5 from 2022. For the remaining 9 events, DP-305423, MON87705, MON87769, CV127, DAS-68416, DAS-81419, GMB151, MON87751, and SYHT0H2, no amplification was detected in any sample. Although it was difficult to simply compare the Cq values obtained because of the differences in the performance of each primer and probe, it was generally considered that lower Cq values generally indicated higher copy numbers for the target sequence, suggesting relatively higher comingling. The comingling levels of MON89788 were higher than those of RRS in all samples. RRS, the first generation of glyphosate-resistant soybean, went off-patent in 2015 and has since been gradually phased out from seed stocks.^30^

**Table 3.** List of Cq* values obtained from non-identity-preserved samples in the United States.

	2021										2022									
GM event	Sample no.										Sample no.									
	N1–1	N1–2	N2–1	N2–2	N3–1	N3–2	N4–1	N4–2	N5–1	N5–2	N1–1	N1–2	N2–1	N2–2	N3–1	N3–2	N4–1	N4–2	N5–1	N5–2
RRS	31.63	31.60	–	–	33.03	33.45	31.95	31.89	31.68	31.37	–	–	33.54	33.54	–	–	35.43	34.64	34.08	33.84
MON89788	24.15	24.17	24.45	24.45	24.80	24.95	23.99	23.87	24.41	24.25	25.38	26.12	25.45	25.43	24.38	24.24	26.04	24.96	25.75	26.09
A2704–12	–	–	–	–	–	–	36.51	–	–	–	–	–	–	–	–	–	34.48	33.35	–	–
A5547–127	26.19	26.32	25.12	25.18	25.48	25.55	25.66	25.61	26.13	25.86	26.32	27.3	26.85	26.74	26.02	25.90	27.71	26.65	27.07	27.41
DP-305423	–	–	–	–	–	–	–	–	–	–	–	–	–	–	–	–	–	–	–	–
MON87701	–	–	–	–	–	–	–	–	–	–	–	–	–	–	37.55	37.63	–	–	–	–
MON87705	–	–	–	–	–	–	–	–	–	–	–	–	–	–	–	–	–	–	–	–
MON87769	–	–	–	–	–	–	–	–	–	–	–	–	–	–	–	–	–	–	–	–
MON87708	24.70	24.86	24.74	24.85	25.24	25.41	24.33	24.29	24.97	24.83	25.72	26.74	25.85	25.79	24.73	24.70	26.21	25.29	26.18	26.33
CV127	–	–	–	–	–	–	–	–	–	–	–	–	–	–	–	–	–	–	–	–
DAS-68416	–	–	–	–	–	–	–	–	–	–	–	–	–	–	–	–	–	–	–	–
DAS-44406	25.99	26.02	25.21	25.26	24.71	24.73	26.93	26.73	25.60	25.64	25.81	26.37	25.31	25.14	24.57	24.66	25.99	25.26	25.27	25.36
DAS-81419	–	–	–	–	–	–	–	–	–	–	–	–	–	–	–	–	–	–	–	–
FG72	27.01	27.02	28.15	28.06	30.61	30.16	–	–	28.84	28.62	–	–	–	–	33.23	33.05	37.35	35.96	34.54	34.60
GMB151	–	–	–	–	–	–	–	–	–	–	–	–	–	–	–	–	–	–	–	–
MON87751	–	–	–	–	–	–	–	–	–	–	–	–	–	–	–	–	–	–	–	–
SYHT0H2	–	–	–	–	–	–	–	–	–	–	–	–	–	–	–	–	–	–	–	–
Le1	24.40	24.30	24.03	24.11	24.13	24.07	24.18	24.17	24.41	24.05	23.92	24.08	24.44	24.40	24.42	24.32	26.14	25.36	25.67	25.64

*Cq : quantification cycle for real-time PCR analysis.

### Kernel-Based Detection Analysis for Identification of Stacked Genetically Modified Varieties

**Figure 1.**
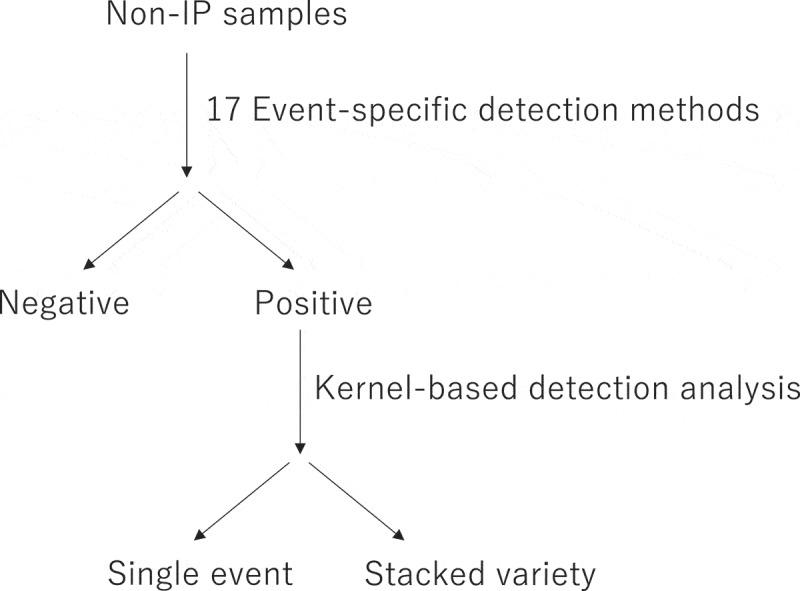
Schematic workflow of the GM soybean detection strategy for non-ip samples.

To determine whether the detected GM soybean events were stacked in the non-IP samples, we collected 48–53 kernels from each sample in 2021 and 2022 and performed individual kernel detection analyses targeting 4 GM events, MON89788, MON87708, A5547–127, and DAS-44406 (Figure 1). For this analysis, because it was a single kernel, the mixing level would be nearly 100% if the kernel was a GM. We used purified DNA as the template, and it was expected that the Cq values would be approximately the same level as the positive control (23–25) in the case of GMs, and that the Cq value would not be obtained in the case of non-GM kernel. However, during the analysis, several kernels had unexpectedly high Cq values of approximately 35–40. Table S3 shows the Cq values obtained in sample N3. The cause of these high Cq values was unclear, and we speculated the effect of DNA from other kernel sources on the surface when conducting individual kernel detection. Then, we removed the seed coats from the kernels, and DNA was extracted separately from the seed coats and internal seed materials (Figure S1). Fourteen kernels were collected and analyzed. High Cq values were frequently observed in DNA extracted from seed coats, and were rarely detected inside the seeds, even in the same samples (Table S4). These findings indicated that kernels that showed high Cq values were GM negative seeds, which were created when a powder from other GM seeds attached to the surfaces of the tested samples. Based on these results, we set a Cq threshold of 35 or higher to classify seeds as GM-negative.

**Figure 2.**
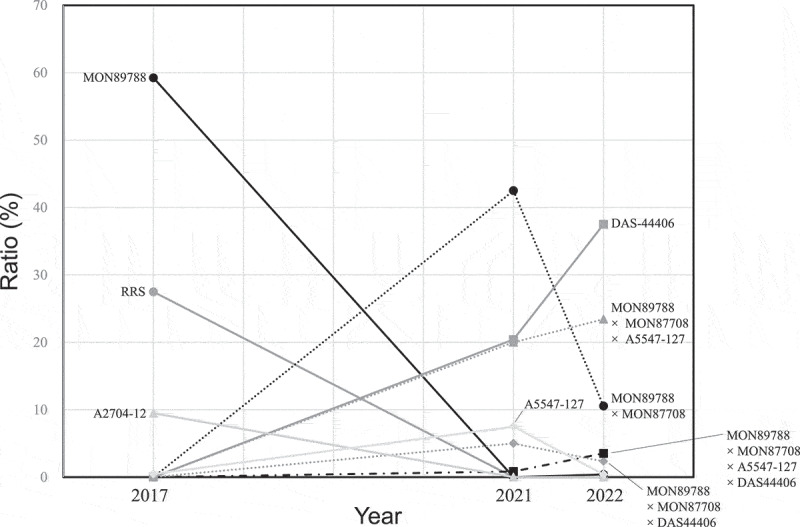
Changes in the ratio of GM soybean varieties from 2017 to 2022. The data in 2017 was cited from Soga et al. Biol Pharm Bull. 43:1259–1266.

The results of the kernel-based detection analyses are listed in Table 4. In the 2021 samples, the most frequently detected GM soybean variety was the stacked variety MON89788 × MON87708 (42.5%), followed by the single event DAS-44406 (20.4%), the triple stacked variety MON89788 × MON87708 × A5547–127 (20%), the single event A 5547–127 (7.5%), and the triple stacked variety MON89788 × MON87708 × DAS-44406 (5%). MON89788 × MON87708 accounted for the majority and single events of DAS-44406 and A5547–127 were present in all samples. In the 2022 samples, the ratio of DAS-44406 single event increased to 37.5%, followed by MON89788 × MON87708 × A5547–127 (23.4%), MON89788 × MON87708 (10.5%), MON89788 × MON87708 × A5547–127 × DAS-44406 (3.5%) varieties. It appears that nearly all detected MON89788 and MON87708 in these samples were stacked varieties containing both events, although the number of investigated kernels and samples was limited. We previously analyzed the ratios of GM soybean varieties of non-IP samples from 2017 using kernel-based inspections.^11^ The results of the 2017 samples were added to this study, and changes in the ratios of GM soybean varieties in 2017, 2021, and 2022 were shown in Figure 2. GM soybeans, containing MON89788, were largely detected as single events in 2017, whereas the proportion of stacked varieties containing MON89788 increased in 2021. Multi-stacked varieties containing MON89788 × MON87708, such as MON89788 × MON87708 × A5547–127 further increased by 2022. In contrast, the ratio of single event of DAS-44406 is increasing considerably from 2021 onwards. These trends may reflect an increase in GM soybean varieties containing multiple herbicide-tolerant traits such as MON89788 × MON87708, which confers tolerance to glyphosate and dicamba, and DAS-44406, which confers tolerance to 2, 4-D, glyphosate, and glufosinate.

**Table 4.** Summary of individual kernel-based detection analyses from non-identity-preserved samples.

	2021							2022						
GM variety	Sample							Sample						
	N1	N2	N3	N4	N5	Total	（%）	N1	N2	N3	N4	N5	Total	（%）
MON89788	0	0	0	0	0	**0**	**0**	0	0	0	1	0	**1**	**0.4**
MON87708	0	0	0	0	0	**0**	**0**	0	1	0	0	0	**1**	**0.4**
A5547–127	3	4	5	3	3	**18**	**7.5**	0	0	0	1	0	**1**	**0.4**
DAS-44406	2	12	17	6	12	**49**	**20.4**	11	15	18	26	26	**96**	**37.5**
MON89788 × MON87708	30	10	16	24	22	**102**	**42.5**	0	2	14	7	4	**27**	**10.5**
MON89788 × MON87708 × A5547–125	0	14	10	13	11	**48**	**20**	8	12	19	14	7	**60**	**23.4**
MON89788 × MON87708 × DAS-44406	9	3	0	0	0	**12**	**5**	0	0	0	0	6	**6**	**2.3**
MON89788 × MON87708 × A5547–27 × DAS-44406	1	1	0	0	0	**2**	**0.8**	0	0	0	0	9	**9**	**3.5**
A5547–127 × MON87708	0	0	0	0	0	**0**	**0**	0	0	0	2	0	**2**	**0.78**
DAS-44406 × A5547–127	2	0	0	0	0	**2**	**0.8**	0	0	0	0	0	**0**	**0**
Others	1	4	0	2	0	**7**	**2.9**	32	19	2	0	0	**53**	**20.7**

## Discussion

GM crops, including GM soybeans has been developed continuously in recent years. However, limited information is available on which varieties are primarily cultivated and distributed in the US, which has the largest number of approved GM soybean varieties and the most extensive area of GM crop cultivation. This is particularly relevant for non-IP samples, as these are often intended for export to countries such as Japan, where knowledge of the predominant GM soybean events or stacked varieties in the US cultivation would be useful. In 2017, an investigation into the distribution of non-IP samples from the US reported that over 95% of these samples consisted of single events including RRS, MON89788, and A2704–12.^11^ In the Japanese standard method, specific detection methods for these 3 events have been adopted,^10^ and it was sufficient for the labeling system for GM soybean at that time.

More recently, we evaluated maize distribution samples, targeting both IP and non-IP samples like soybean. It was revealed that the GM maize events detected in those samples were adequately covered by the Japanese standard method.^31^

In this study, we re-investigated soybean samples from the US and Canada domestically distributed in 2021 and 2022. In the single kernel analysis, single events such as RRS and MON89788 had largely been replaced by stacked varieties containing these events. Consequently, it is difficult to determine whether a processed food product contains single events or stacked varieties. Under the Japanese standard method, quantitative detection methods, including kernel-based analysis, are available for seeds but not for processed products. From non-IP samples, it was suggested that certain GM soybeans such as MON87708, DAS-44406, A5547–127, MON87701 and FG72 were not directly detectable with the current method. Among these events, MON87708, DAS-44406, and A5547–127, which were detected in non-IP samples, underwent kernel-based detection analysis. Nearly, all MON87708 were stacked varieties with MON89788, indicating that these stacked GM varieties containing MON87708 were detectable with the MON89788 detection method. If MON87708 were quantified separately from MON89788, it would result in a double quantification of MON89788 and MON87708, suggesting the possibility of overestimating the comingling rate. Therefore, for the stacked varieties containing MON87708, more efficient and accurate results could be achieved by conducting quantitative analysis for MON89788, but not for MON87708. If the prevalence of the single event of MON87708 increases in the future, a specific quantification method for MON87708 will be required. Regular monitoring of distribution samples is therefore important. DAS-44406 and A5547–127 were also detected in all samples. In the case of A5547–127, it tends to contain more stacked varieties than single event (Table 4). A5547–127, as a single event, accounted for 7.5% of the samples from 2021, but this ratio dropped significantly to 0.4% in the samples from 2022. Moreover, A5547–127 contains the P35S sequence, indicating that screening detection targeting P35S is applicable. From these results, a specific detection method for A5547–127 is not currently indispensable. In contrast, DAS-44406 does not contain common sequences such as P35S, TNOS, or EPSPS, which have been used as targets for screening detection. The single event DAS-44406 was detected in all non-IP sample lots and was the second most abundant event in the 2021 samples and was the most abundant in the 2022 samples. Additionally, DAS-44406 was detected in some IP sample lots although the event is not detectable with the current Japanese standard method. Therefore, an event-specific or screening detection method for DAS44406 will be necessary.

## Supplementary Material

Supplemental Material
