# Supplementary material for: Distribution status of genetically modified soybeans from the United States and Canada to Japan in 2021 and 2022
Source: GM Crops Food. 2024 Dec 26;16(1):116–25. doi: 10.1080/21645698.2024.2444048 (PMC11702959; doi:10.1080/21645698.2024.2444048)
Supplement: Supplemental Material [file KGMC_A_2444048_SM9299.doc]

Figure S1. Representative images of the seed coat and interior of the soybean seeds.

Table S1. GM soybean events analyzed in this study

| Event name | Trait | Resisted herbicide | Year of approval in Japan |
| --- | --- | --- | --- |
| RRS | Herbicide tolerance | Glyphosate | 2001 |
| MON89788 | Herbicide tolerance | Glyphosate | 2007 |
| A2704-12 | Herbicide tolerance | Glufosinate | 2002 |
| A5547-127 | Herbicide tolerance | Glufosinate | 2002 |
| DP-305423 | Herbicide tolerance,  Modified oil/fatty acid | Sulfonylurea | 2010 |
| MON87701 | Insect resistance |  | 2011 |
| MON87705 | Herbicide tolerance,  Modified oil/fatty acid | Glyphosate | 2012 |
| MON87769 | Modified oil/fatty acid |  | 2014 |
| MON87708 | Herbicide tolerance | Glyphosate, Dicamba | 2013 |
| CV127 | Herbicide tolerance | Sulfonylurea | 2012 |
| DAS-68416 | Herbicide tolerance | Glufosinate, 2,4-D | 2014 |
| DAS-44406 | Herbicide tolerance | Glufosinate, Glyphosate, 2,4-D | 2014 |
| DAS-81419 | Herbicide tolerance,  Insect resistance | Glufosinate | 2014 |
| FG72 | Herbicide tolerance | Glyphosate, Isoxaflutole | 2016 |
| GMB151 | Nematode resistance,  Herbicide tolerance | Hydroxyphenylpyruvate dioxygenase inhibitor | 2022 |
| MON87751 | Insect resistance |  | 2016 |
| SYHT0H2 | Herbicide tolerance | Glufosinate, Mesotrione | 2016 |

Table S2. List of Cq* values obtained from identity-preserved samples from Canada

|  | **2021** | | | | | | | | | | **2022** | | | | | | | | | |
| --- | --- | --- | --- | --- | --- | --- | --- | --- | --- | --- | --- | --- | --- | --- | --- | --- | --- | --- | --- | --- |
| **GM event** | Sample | | | | | | | | | | Sample | | | | | | | | | |
|  | I1-1 | I1-2 | I2-1 | I2-2 | I3-1 | I3-2 | I4-1 | I4-2 | I5-1 | I5-2 | I1-1 | I1-2 | I2-1 | I2-2 | I3-1 | I3-2 | I4-1 | I4-2 | I5-1 | I5-2 |
| **RRS** | - | - | - | - | - | - | - | - | - | - | - | - | - | - | - | - | - | - | - | - |
| **MON89788** | 32.35 | 33.39 | - | - | - | - | - | - | - | - | - | - | - | - | - | - | - | - | - | - |
| **A2704-12** | - | - | - | - | - | - | - | - | - | - | - | - | - | - | - | - | - | - | - | - |
| **A5547-127** | - | - | - | - | - | - | - | - | - | - | - | - | - | - | - | - | - | - | - | - |
| **DP-305423** | - | - | - | - | - | - | - | - | - | - | - | - | - | - | - | - | - | - | - | - |
| **MON87701** | - | - | - | - | - | - | - | - | - | - | - | - | - | - | - | - | - | - | - | - |
| **MON87705** | - | - | - | - | - | - | - | - | - | - | - | - | - | - | - | - | - | - | - | - |
| **MON87769** | - | - | - | - | - | - | - | - | - | - | - | - | - | - | - | - | - | - | - | - |
| **MON87708** | - | - | - | - | - | - | - | - | - | - | - | - | - | - | - | - | - | - | - | - |
| **CV127** | - | - | - | - | - | - | - | - | - | - | - | - | - | - | - | - | - | - | - | - |
| **DAS-68416** | - | - | - | - | - | - | - | - | - | - | - | - | - | - | - | - | - | - | - | - |
| **DAS-44406** | - | - | - | - | - | - | - | - | - | - | - | - | - | - | - | - | - | - | - | - |
| **DAS-81419** | - | - | - | - | - | - | - | - | - | - | - | - | - | - | - | - | - | - | - | - |
| **FG72** | - | - | - | - | - | - | - | - | - | - | - | - | - | - | - | - | - | - | - | - |
| **GMB151** | - | - | - | - | - | - | - | - | - | - | - | - | - | - | - | - | - | - | - | - |
| **MON87751** | - | - | - | - | - | - | - | - | - | - | - | - | - | - | - | - | - | - | - | - |
| **SYHT0H2** | - | - | - | - | - | - | - | - | - | - | - | - | - | - | - | - | - | - | - | - |
| **Le1** | 24.14 | 24.17 | 24.27 | 24.17 | 24.23 | 24.32 | 24.17 | 24.09 | 24.10 | 24.22 | 24.28 | 24.20 | 24.03 | 24.11 | 24.13 | 24.07 | 24.18 | 24.17 | 24.41 | 24.05 |

*Cq : quantification cycle for real-time PCR analysis

Table S3. Results of individual kernel-based detection analysis of non-identity-preserved samples from the United States

| Sample | US3-1 | US3-2 | US3-3 | US3-4 | US3-5 | US3-6 | US3-7 | US3-8 | US3-9 | US3-10 | US3-11 | US3-12 | US3-13 | US3-14 | US3-15 | US3-16 |
| --- | --- | --- | --- | --- | --- | --- | --- | --- | --- | --- | --- | --- | --- | --- | --- | --- |
| Le1 | 25.13 | 24.92 | 25.26 | 24.60 | 25.07 | 24.68 | 24.51 | 24.60 | 24.72 | 24.34 | 24.46 | 24.67 | 24.82 | 24.40 | 24.55 | 26.00 |
| MON89788 | - | 23.91 | - | 23.77 | - | 24.03 | 23.88 | 23.96 | 24.01 | 23.71 | 23.62 | 23.83 | 37.03 | 23.85 | - | 24.78 |
| MON87708 | - | 23.95 | 38.72 | 23.95 | 38.94 | 23.90 | 23.95 | 23.96 | 23.96 | 23.58 | 23.56 | 23.85 | 36.95 | 23.98 | - | 24.94 |
| DAS-44406 | 24.13 | 40.15 | 24.24 | - | 24.31 | - | 37.38 | - | 39.24 | - | - | - | 23.98 | - | 38.64 | 35.14 |
| A5547-127 | - | - | - | 23.76 | 39.09 | - | 23.85 | - | - | - | 23.40 | - | 36.10 | - | 23.54 | 24.35 |
|  | | | | | | | | | | | | | | | | |
| Sample | US3-17 | US3-18 | US3-19 | US3-20 | US3-21 | US3-22 | US3-23 | US3-24 | US3-25 | US3-26 | US3-27 | US3-28 | US3-29 | US3-30 | US3-31 | US3-32 |
| Le1 | 25.36 | 24.55 | 24.69 | 24.56 | 25.09 | 25.36 | 24.91 | 24.30 | 24.53 | 25.15 | 24.34 | 24.95 | 24.70 | 24.78 | 24.60 | 24.91 |
| MON89788 | 24.71 | 23.95 | - | 23.92 | 24.49 | - | 24.11 | 23.86 | 39.26 | 24.22 | 35.98 | 24.41 | 24.07 | - | 38.05 | - |
| MON87708 | 24.57 | 23.93 | - | 23.99 | 24.53 | 37.23 | 23.90 | 23.93 |  | 24.17 | 36.63 | 24.30 | 24.11 | 39.01 | 38.99 | - |
| DAS-44406 | - | 38.33 | 24.02 | 37.96 | - | 24.83 | 36.71 | - | 38.83 | - | 23.85 | 38.96 | - | 24.01 | 23.92 | 24.22 |
| A5547-127 | 24.90 | 39.84 | - | 37.14 | - | - | 37.29 | - | 23.50 | - | 36.28 | 24.16 | 24.23 | 39.55 | 38.39 | - |
|  | | | | | | | | | | | | | | | | |
| Sample | US3-33 | US3-34 | US3-35 | US3-36 | US3-37 | US3-38 | US3-39 | US3-40 | US3-41 | US3-42 | US3-43 | US3-44 | US3-45 | US3-46 | US3-47 | US3-48 |
| Le1 | 24.46 | 24.48 | 24.88 | 24.54 | 26.96 | 26.31 | 24.63 | 25.08 | 24.64 | 24.83 | 25.50 | 25.10 | 24.44 | 24.42 | 24.81 | 25.88 |
| MON89788 | 23.88 | - | - | - | 25.11 | - | 23.81 | 24.57 | - | 24.08 | 39.38 | 40.28 | 38.19 | 37.50 | - | 25.12 |
| MON87708 | 23.72 | 38.82 | - | - | 25.57 | 38.77 | 23.90 | 24.49 | - | 23.92 | - | - | 37.85 | 38.04 | - | 25.23 |
| DAS-44406 | 36.77 | 24.06 | 24.00 | - | 40.66 | 25.16 | 38.58 | 38.72 | 23.67 | - | 24.65 | 38.73 | 37.87 | 23.84 | 24.06 | - |
| A5547-127 | 23.73 | - | - | 23.73 | 24.88 | - | - | 24.03 | 40.00 | 40.00 | - | 24.43 | 23.67 | 37.99 | - | - |

Relatively high Cq values are underlined.

Table S4. Results of individual kernel-based detection analyses using the seed coat or inside of the seed of non-identity-preserved samples from the United States

|  | Sample | 1 | 2 | 3 | 4 | 5 | 6 | 7 | 8 | 9 | 10 | 11 | 12 | 13 | 14 |
| --- | --- | --- | --- | --- | --- | --- | --- | --- | --- | --- | --- | --- | --- | --- | --- |
| Seed coat | Le1 | 24.71 | 24.67 | 24.89 | 25.00 | 24.74 | 24.65 | 26.39 | 24.66 | 24.97 | 24.54 | 24.52 | 24.91 | 25.46 | 25.34 |
|  | MON89788 | 38.12 | 36.96 | 24.31 | 24.54 | 38.81 | - | 39.20 | - | 37.61 | - | 24.07 | 39.97 | 38.87 | 38.15 |
|  | MON87708 | 37.47 | 35.68 | 23.56 | 23.71 | 36.96 | - | 37.90 | 38.37 | 36.82 | 39.74 | 23.37 | 38.70 | 36.70 | 37.97 |
|  | DAS-44406 | 23.73 | 37.97 | 38.25 | 37.37 | 38.08 | 23.91 | 36.25 | 23.73 | 23.84 | 38.57 | - | 38.29 | 24.37 | 38.82 |
|  | A5547-127 | 38.39 | 24.19 | - | 24.50 | 24.09 | 39.49 | 25.75 | - | 38.47 | 24.09 | 39.78 | 23.98 | 37.14 | 24.56 |
| Inside of seed | Le1 | 24.65 | 24.89 | 25.90 | 24.60 | 24.70 | 24.68 | 26.16 | 26.33 | 24.77 | 25.56 | 25.10 | 24.16 | 25.41 | 24.70 |
|  | MON89788 | - | - | 24.74 | 23.74 | - | - | - | - | 37.71 | - | 24.26 | - | - | - |
|  | MON87708 | - | - | 25.03 | 24.00 | - | - | - | - | - | - | 24.46 | - | - | - |
|  | DAS-44406 | 23.93 | - | - | - | - | 24.10 | - | 25.89 | 23.93 | - | - | - | 24.53 | - |
|  | A5547-127 | - | 23.95 | - | 23.92 | 23.84 | - | 24.99 | - | - | 24.97 | - | 23.64 | - | 24.05 |
| GM variety | | DAS-44406 | A5547-127 | MON89788 × MON87708 | MON89788 ×  MON87708 ×  A5547-125 | A5547-127 | DAS-44406 | A5547-127 | DAS-44406 | DAS-44406 | A5547-127 | MON89788 × MON87708 | A5547-127 | DAS-44406 | A5547-127 |

Relatively high Cq values are underlined.
